# Euthanasia and physician-assisted suicide in people with intellectual disabilities and/or autism spectrum disorders: investigation of 39 Dutch case reports (2012–2021)

**DOI:** 10.1192/bjo.2023.69

**Published:** 2023-05-23

**Authors:** Irene Tuffrey-Wijne, Leopold Curfs, Sheila Hollins, Ilora Finlay

**Affiliations:** Faculty of Health, Science, Social Care and Education, Kingston University London, Kingston upon Thames, UK; Governor Kremers Centre, Maastricht University Medical Centre, Maastricht, The Netherlands; Department of Pharmacology, Radiology, Oncology & Palliative Medicine, Cardiff University, Cardiff, UK

**Keywords:** Euthanasia, physician-assisted suicide, assisted dying, intellectual disability, autism spectrum disorders

## Abstract

**Background:**

Euthanasia review committees (Regionale Toetsingscommissies Euthanasie, RTE) scrutinise all Dutch cases of euthanasia and physician-assisted suicide (EAS) to review whether six legal ‘due care’ criteria are met, including ‘unbearable suffering without prospect of improvement’. There are significant complexities and ethical dilemmas if EAS requests are made by people with intellectual disabilities or autism spectrum disorders (ASD).

**Aims:**

To describe the characteristics and circumstances of people with intellectual disabilities and/or ASD who were granted their EAS request; investigate the main causes of suffering that led to the EAS request; and examine physicians’ response to the request.

**Method:**

The online RTE database of 927 EAS case reports (2012–2021) was searched for patients with intellectual disabilities and/or ASD (*n* = 39). Inductive thematic content analysis was performed on these case reports, using the framework method.

**Results:**

Factors directly associated with intellectual disability and/or ASD were the sole cause of suffering described in 21% of cases and a major contributing factor in a further 42% of cases. Reasons for the EAS request included social isolation and loneliness (77%), lack of resilience or coping strategies (56%), lack of flexibility (rigid thinking or difficulty adapting to change) (44%) and oversensitivity to stimuli (26%). In one-third of cases, physicians noted there was ‘no prospect of improvement’ as ASD and intellectual disability are not treatable.

**Conclusions:**

Examination of societal support for suffering associated with lifelong disability, and debates around the acceptability of these factors as reasons for granting EAS, are of international importance.

## Euthanasia and physician-assisted suicide in The Netherlands

The Netherlands’ 2002 Termination of Life on Request and Assisted Suicide Act made it legally possible to perform euthanasia (where a physician administers a fatal dose of a drug to the patient at his or her express request) or physician-assisted suicide (where the physician supplies the drug, but the patient administers it), provided that six ‘due care’ criteria are met (see Supplementary File 1 available at https://dx.doi.org/10.1192/bjo.2023.69). Physicians who carry out euthanasia and physician-assisted suicide (EAS) must report each case; these reports are examined by a euthanasia review committee (Regionale Toetsingscommissie Euthanasie, RTE), tasked with adjudicating whether the requirements of due care had been observed and met. One of the due care criteria, and the focus of this paper, is that ‘the patient’s suffering is unbearable, with no prospect of improvement’. In their EAS report, physicians must explain what the suffering consisted of, why they were convinced it was unbearable and how they came to the conclusion that there was no prospect of improvement.^1^

In 2021 in The Netherlands, EAS accounted for 7666 deaths (4.5% of all deaths).^2^ The vast majority of EAS requests granted by physicians (89%) were for patients whose ‘unbearable suffering’ was caused by somatic conditions, predominantly cancer. However, a life-limiting condition is not a prerequisite for granting an EAS request. Dutch law requires the ‘unbearable suffering’ to have a medical basis, but this can be either somatic or psychiatric. It permits EAS for unbearable suffering caused by psychiatric conditions, dementia, various geriatric syndromes, chronic pain syndromes or genetic conditions. Although the percentage of these EAS requests may be low, the number of deaths through EAS for reasons other than terminal illness is increasing and not insignificant.

## Intellectual disability and autism spectrum disorder

People fall within the definition of intellectual disability if all of the following apply: (a) limitations (‘deficits’) in intellectual functioning (IQ score ≤70); (b) impairments in adaptive and/or social functioning; (c) these limitations occur before adulthood and are lifelong. Autism spectrum disorder (ASD) is a complex and usually lifelong developmental disorder, characterised by persistent difficulties in social communication and social interaction across multiple contexts. This can include deficits in social-emotional reciprocity, in non-verbal communicative behaviours and in developing, maintaining and understanding relationships. Although ASD is common among people with intellectual disabilities, not all people with ASD have an intellectual disability; they may have average or above-average intelligence.^3^

Estimates of prevalence are around 1–2% of the population for intellectual disability^4,5^ and 1–2% for ASD.^6^

## Euthanasia and physician-assisted suicide in people with intellectual disability and/or ASD

People with intellectual disability or ASD are not excluded from making, and being granted, an EAS request. Their right to do so is in line with the UN Convention on the Rights of Persons with Disabilities.^7^ The significant complexities and ethical dilemmas in such cases were highlighted in an examination of nine cases of EAS granted to people with intellectual disabilities and/or ASD; only two of these had progressive and life-limiting somatic conditions. Assessment of suffering was particularly difficult for patients with lifelong disability.^8^

## Aims

The aims of this study are to (a) describe the characteristics and circumstances of people with intellectual disabilities or ASD who were granted their EAS request; (b) investigate the main causes of suffering and the factors associated with or contributing to the experience of unbearable suffering that led to the EAS request; and (c) examine how physicians assessed and responded to the EAS request.

This paper follows the Standards for Reporting Qualitative Research (SRQR) reporting guidelines.

## Method

### Researcher characteristics

The authors of this paper are experts in the fields of intellectual disability and palliative care. We come from The Netherlands and the UK, which have divergent legal frameworks as regards EAS. Our aim is not to present or promote a common ethical perspective, but to contribute to the international debate by presenting the findings of a rigorously conducted study of public data and our assessment of the implications. The first and second authors are native Dutch speakers and fluent English speakers.

### Sample

Between 2012 and 2021, the Dutch RTEs received 59 996 notifications of EAS; case summaries of 927 of these (1.5%) are included (in Dutch) in a searchable open access database on the RTE website (https://www.euthanasiecommissie.nl), with the specific aim of showing how the committee applied and interpreted the legal due care criteria and how they dealt with particular challenges. No pre-2012 case reports are available.

We searched the database for case reports involving people who had intellectual disability and/or ASD by using the following Dutch keywords: *verstandelijk* [mental], *verstandelijke beperking* [mental/intellectual disability], *intellectuele beperking* [intellectual disability], *zwakbegaafd* [mentally disabled], *verminderde intelligentie* [low intelligence], *autisme* [autism], *ASS* [autism spectrum disorder], Asperger. Case reports where the person did not have an intellectual disability or ASD (for example, they had cognitive limitations due to dementia, or they had been assessed for ASD but found not to have it) were excluded from the results. This left a total of 39 relevant case reports for inclusion in the study; reportedly, 15 people had intellectual disability, 20 had ASD and 4 had both intellectual disability and ASD (Supplementary File 2).

The 39 case reports were between 852 words and 4436 words long (median 1907 words), describing the physician’s written reports of the nature of the patient’s suffering, possible alternatives to EAS, discussions between physicians and patient, the EAS request, the consultations with other physicians (including second independent opinion), how the EAS was carried out, any further verbal explanations requested from the physician by the RTE, and the considerations and verdict of the RTE.

### Data analysis

We performed inductive thematic analysis of the data, using the framework method^9^ supported by Nvivo software (version 12). The steps were as follows. (1) Familiarisation: the first author (I.T.-W.) read and re-read all 39 case reports; the second author (L.C.) read a selection of case reports. The first author translated a summary of each case into English, which was read by all four authors. (2) Initial inductive coding: reading the cases line by line, the first author applied a label (‘code’) to all possible factors and contributors, resulting in a total of 353 coded segments. (3) Developing a coding framework: the first and second authors discussed the codes and developed a detailed coding framework consisting of 22 categories. The framework was discussed and agreed with all four authors. (4) Final coding: the first author coded all 39 full documents into the agreed framework. This allowed for frequency tabulation as well as descriptive overviews of the contributing factors. (5) Final categorisation: the main causes of suffering were categorised according to whether they related to characteristics of ASD/intellectual disability, somatic conditions, psychiatric conditions or a combination of these. Each case summary was re-read and discussed with all four authors to reach consensus on final categorisation.

### Ethics statement

Both the Kingston University Research Ethics Committee (London, UK) and the Legal Council of University Hospital Maastricht (The Netherlands) confirmed that our study was exempt from ethical review, so no informed consent was required. We used anonymised data that are publicly available on the RTE website. The content of the RTE website is subject to a Creative Commons Zero (CC0) declaration, which means that re-use is permitted (www.euthanasiecommissie.nl/copyright). The RTE website states that they select anonymised cases for publication, based on their relevance to the development of standards and their importance in terms of public and societal interest.^10^ We discussed our study with a lawyer who is responsible for ethical issues involved in the use of the RTE database. They highlighted that a committee appointed by the Dutch government, which oversees ethical issues around use of the database, has recommended that the published data may be used or commented on freely and without need for further ethical approvals, provided that they are not combined with any other data-sets (such as death records) that might compromise anonymity. Our study protocol involved scrutiny of the publicly available data only, and therefore the lawyer confirmed that as these have already been subject to Dutch government considerations, further ethical approvals were not required.

## Results

### Patient characteristics and circumstances

An overview of patient characteristics and circumstances is given in Table 1.

**Table 1:** Patient characteristics and social circumstances (*n* = 39)

Characteristic	Number of cases	Proportion of cases
Intellectual disability/autism spectrum disorder		
Intellectual disability only	19	49%
Autism spectrum disorder only	24	62%
Both intellectual disability and autism spectrum disorder	4	10%
Gender		
Male	19	49%
Female	20	51%
Age, years		
18–29	5	13%
30–39	7	18%
40–49	6	15%
50–59	5	13%
60–69	4	10%
70–79	7	18%
80–89	3	8%
≥90	2	5%
Somatic conditions	26	67%
Arthritis/osteoporosis	7	18%
Cancer	3	8%
Chronic fatigue syndrome	1	3%
Diabetes	3	8%
Epilepsy	2	5%
Fractures	2	5%
Gallstones	1	3%
Heart and circulatory disorders	5	13%
Kidney and bladder disorders	2	5%
Lungs	5	13%
Multiple geriatric syndromes	3	8%
Paralysis	1	3%
Parkinson's disease	2	5%
Sensory (hearing, sight)	7	18%
Medically unexplained physical symptoms	4	10%
Spina bifida	1	3%
Tinnitus	2	5%
Tourette syndrome	1	3%
Psychiatric conditions	25	64%
Attention-deficit hyperactivity disorder	1	3%
Bipolar disorder	1	3%
Borderline personality disorder	8	21%
Depression	12	31%
Dissociative disorder	1	3%
Obsessive–compulsive disorder	10	26%
Paranoia	3	8%
Personality disorder	6	15%
Psychosis	8	21%
Psychosocial problems	3	8%
Post-traumatic stress disorder	4	10%
Pyromania	1	3%
Somatoform disorder	2	5%
Other characteristics		
Suicidal	17	44%
Trauma (childhood)	7	18%
Trauma (adult)	8	21%
Substance misuse	5	13%
Social circumstances		
Supported living/residential care	8	21%
Previous psychiatric in-patient episodes	16	41%
Family mentioned in report	12	31%
Family involved in EAS process	6	15%

EAS, euthanasia and physician-assisted suicide.

#### Characteristics

Of the 39 cases, 19 patients (49%) had intellectual disability and 24 (62%) had ASD; of these, 4 (10%) had both intellectual disability and ASD. All age groups were represented, with 18 patients (46%) younger than 50 when they died. A very wide range of diagnosed somatic conditions were mentioned for 26 patients (67%), with most having more than one condition, but no single somatic condition was predominant. Cancer (the predominant cause of suffering in 61% of all EAS cases in The Netherlands) was mentioned for 3 patients (8%). Psychiatric conditions were mentioned for 25 patients (64%). Of these, depression was the most common (31% of patients), followed by obsessive–compulsive disorder, borderline personality disorder and psychosis (26%, 21% and 21% respectively).

Other notable characteristics included suicidal thoughts or suicide attempts (44%), childhood trauma, including abuse and neglect (18%), adult trauma, including life events such as bereavements (21%), and substance misuse (13%).

#### Circumstances

Descriptions of the patients’ social circumstances were limited. Although past psychiatric in-patient episodes (in some cases frequent) were mentioned in 41% of cases, current living situations or social support structures were not always made clear, apart from 8 patients (21%) who were in supported living or residential settings. More than two-thirds of the case reports made no mention of the patient’s family or other significant people in their lives. Of the 12 case reports (31%) where family was mentioned, only half (15%) indicated that EAS discussions had involved family or that family was present at the death. The others mentioned the existence of family just briefly, such as:

‘The patient was unable to make friends and had become isolated, including within her own family’ (2017–80, female, age 18–30, ASD).

### Main causes of suffering

The main causes of suffering that led to the EAS requests are summarised in Table 2. Full case examples are given in the Appendix below, to illustrate the different factors contributing to the patients’ experience of unbearable suffering.

**Table 2:** Main cause of suffering (*n* = 39)

Main cause of suffering	Number of cases (intellectual disability/ASD/intellectual disability and ASD)	Proportion of cases	Case example (see Appendix)
Factors associated with:			
Intellectual disability/ASD alone	8 (1/6/1)	21%	2018–24
Intellectual disability/ASD, triggered by somatic conditions	8 (1/5/2)	21%	2020–114
Combination of intellectual disability/ASD and psychiatric conditions	8 (1/6/1)	21%	2020–11
Somatic conditions	6 (6/0/0)	15%	2019–94
Psychiatric conditions	6 (3/3/0)	15%	2020–136
Combination of somatic and psychiatric conditions	3 (3/0/0)	8%	2020–113

ASD, autism spectrum disorder.

In eight cases (21%), the only causes of suffering described were factors directly associated with intellectual disability or ASD. Typically, these people were unable to live with the characteristics of ASD/intellectual disability and could not cope with the world:

‘As he had never been able to keep up with society, he had become insecure, with recurring depression. Due to his intellectual disability, he felt a great pressure of the world on him which he could not handle. His autistic traits made it increasingly difficult for him to cope with changes around him’ (2020–27, male, 70s, intellectual disability and ASD).

In eight cases (21%), ASD or intellectual disability made it difficult to cope with non-life-threatening somatic symptoms or physical decline, such as age-related conditions or symptoms (*n* = 5), tinnitus (*n* = 2) or curable cancer (*n* = 1):

‘The routine that she had introduced into her life also gave the patient something to hold on to when dealing with a world that was complex for her [ … ] The progressive physical deterioration and the lifelong inability to deal with her environment other than in fixed patterns caused unbearable suffering’ (2018–14, female, 80s, ASD).

In a further eight cases (21%), ASD/intellectual disability was a major contributing factor to the person's inability to cope with their psychiatric condition; or the main causes of suffering were described as a combination of psychiatric conditions and the characteristics associated with ASD or intellectual disability. In one case, there was an additional somatic cause of suffering (chronic fatigue syndrome):

‘The patient mainly suffered from anxiety, compulsive complaints and loneliness due to the limitations that arose from ASD, obsessive–compulsive disorder, acquired brain injury, and personality disorder’ (2020–150, male, 40s, ASD).

In 15 cases (38%), the person's EAS request stemmed from suffering that was not substantially related to their ASD or intellectual disability, but related to psychiatric conditions (*n* = 6), somatic conditions (*n* = 6; all of these were people with intellectual disabilities) or a combination (*n* = 3).

### Factors associated with the experience of unbearable suffering

Table 3 gives a summary of the factors associated with or contributing to the experience of unbearable suffering that led to the EAS request. The following factors were particularly associated with having ASD and/or intellectual disability: social isolation and loneliness, a lack of resilience or coping strategies, lack of flexibility and oversensitivity to stimuli.

**Table 3:** Factors associated with suffering (*n* = 39)

Factor	Number of cases	Proportion of cases
Social isolation/loneliness	30	77%
Physical symptoms	27	69%
Pain	19	49%
Tiredness	13	33%
Sleeping problems	9	23%
Dependence	24	62%
Lack of resilience or coping (with the world, life and/or illness)	22	56%
Poor quality of life	19	49%
Loss of hope	18	46%
Lack of flexibility	17	44%
Difficulty adapting to change	12	31%
Rigid thinking/fixation	10	26%
Oversensitivity to stimuli	10	26%
Loss of control	6	15%
Negative self-image	6	15%

#### Social isolation and loneliness

Over three-quarters of patients described being lonely or socially isolated as a major cause of suffering. This often stemmed from feeling rejected and different from others. For patients with ASD in particular, their difficulty in making or coping with social contacts was a major factor:

‘The patient had felt unhappy since childhood and was persistently bullied because he was just a bit different from others [ … ] [He] longed for social contacts but was unable to connect with others. This reinforced his sense of loneliness. The consequences of his autism were unbearable for him [ … ] The prospect of having to live on in this way for years was an abomination to him and he could not bear it’ (2021–26, male, 20s, ASD)

*‘*The patient suffered from his inability to participate in society [ … ] [He] was not able to live among people, because he was easily overstimulated. This made him isolated’ (2019–22, male, 70s, ASD)

*‘*She suffered from the social isolation that her behaviour had led to. Meetings were disturbed by her shouting. People thought her repulsive and nobody wanted to be near her. She was unable to give her life meaning in any other way’ (2018–12, female, 80s, intellectual disability).

#### Lack of resilience or coping strategies

For more than half of patients (*n* = 22, 56%), difficulty in coping with life or with the world (often described as a lack of resilience) was a major contributor to their EAS request. For some, this had been a lifelong cause of unbearable suffering:

‘Panic and despair were constant companions. The patient felt powerless to function in today’s society and could not be the person he wanted to be, with a job and a family’ (2018–69, male, 50s, intellectual disability and ASD)

‘The patient found the world too complex’ (2019–22, male, 70s, ASD).

For others, somatic or psychiatric deterioration tipped them over the edge, as their coping ability was exceeded:

‘The patient had insufficient strategies to cope with his illness’ (2018–27, male, 70s, intellectual disability)

*‘*The patient could not cope with losing her fixed routines and with her increased dependence. This made her frustrated, desperate and sad’ (2018–14, female, 80s, ASD).

#### Lack of flexibility

Rigid coping strategies, a need to stick to routines, difficulties in considering alternatives, and compulsive behaviours were a major cause of suffering for 17 patients (44%). For them, this lack of flexibility tipped the balance and was a major factor in their EAS request:

‘With her limited thinking abilities, the patient was only focused on the complete removal of the tinnitus. The moment she realised “I will never get rid of it”, her suffering had become hopeless and unbearable for her, and she was only focused on euthanasia’ (2015–83, female, 60s, intellectual disability)

*‘*She rejected help from others because she wanted to keep doing everything herself, following fixed rituals, even when that was barely possible anymore’ (2016–48, female, 90s, intellectual disability and ASD)

‘The independent psychiatrist was of the opinion that the severity of suffering was related to his limited coping mechanisms and flexibility, stemming from ASD, with a fixation on the problem rather than an ability to let go and bear it’ (2020–33, male, 50s, ASD).

#### Oversensitivity to stimuli

Of the ten patients for whom an oversensitivity to stimuli was a cause of suffering leading to their EAS request, nine had ASD. Sensitivity to stimuli was usually described as part of a list of other difficulties. For example, one patient was described as suffering from fits of anger caused by fear, obsessively holding on to routines, overwhelming sadness and unexplained, intractable pain:

‘She also suffered from oversensitivity to stimuli such as noises, temperature or touch [ … ] she was barely resilient due to being easily overstimulated’ (2017–80, female, age 18–30, ASD).

### Physician and RTE response

Physicians had the task of ascertaining that the patients’ suffering was unbearable and had no prospect of improvement. This was not easy. Most cases were highly complex, with multiple factors contributing to the EAS request.

The request was agreed, and EAS carried out, by the patient's own physician (usually the general practitioner) in seven cases (18%) and a psychiatrist or mental health services in five cases (13%). The majority of cases (27, 69%) were assessed and agreed by the Expertisecentrum Euthanasie (Euthanasia Expertise Centre (EEC)), mostly (*n* = 22) because the patient's own physician found the case too complex. (The EEC consists of teams of physicians and nurses who offer EAS to patients whose request was denied by their own physician. If they find that all due care criteria are met, an EEC physician can carry out the EAS.^11^)

#### Assessment of unbearable suffering

In one-third of cases, physicians noted explicitly that ASD and intellectual disability are not treatable and that this was the key consideration in their assessment that there was no prospect of improvement in the patient's suffering:

‘ASD is incurable and its treatment is purely symptomatic’ (2019–99, female, 30s, ASD)

*‘*ASD can be supported, but it can't be treated’ (2020–113, female, 50s, ASD and intellectual disability).

Physicians noted that fundamentally, the patient's suffering stemmed from the limitations of ASD or intellectual disability:

‘Because of this, the patient was unable to build a ‘normal’ life [ … ] The physician thought there was no prospect of improvement. Learning to live with his limitation would be the only option for the patient’ (2020–44, male, 40s, ASD)

*‘*According to [the physician], the huge burden of suffering had to be seen in the context of the patient's limited resilience and coping, which was a result of his limited intelligence and near-absence of the ability to reflect’ (2018–27, male, 70s, intellectual disability).

The fact that treatments had not helped, and would not help, was noted by physicians in 31 cases (79%); this was often strongly influenced by the perceived limitations of ASD or intellectual disability, which led to a lack of flexibility, adaptability and resilience:

‘The patient's personality and her intellectual limitations resulted in an inability to profit from psychological or psychiatric interventions’ (2015–24, female, 60s, intellectual disability)

‘His intellectual disability and affective neglect in childhood had led to insufficient resilience to cope with suffering’ (2018–71, male, 50s, intellectual disability).

Where the suffering consisted solely or significantly of the way ASD or intellectual disability affected the patient, physicians sometimes struggled to apply the EAS due care criteria. In 14 cases (36%) the assessing physician did not think that the criteria for EAS were met:

‘According to [the first consultant], because there was no medical basis for the suffering, not all requirements had been met’ (2018-27, male, 70s, intellectual disability).

In most of those cases, the patient was referred to the EEC for further assessment and/or seen by another consultant, eventually leading to the conclusion that EAS was the right option for the patient.

### RTE verdict

In two cases (2017*–*14 and 2018*–*69) the RTE concluded that the due care criteria had not been met, because of the physician’s failure to seek adequate advice from independent consultants. In case 2018–69 (male, 50s, ASD and intellectual disability) the independent consultant who assessed the patient concluded that, even though the main diagnosis (ASD) was untreatable, there were still options for improving the patient's resilience and for helping him cope better with the death of a parent; therefore, the legal EAS criteria had not been met. The patient’s physician disagreed and carried out the euthanasia. The RTE’s verdict was that the physician should have sought a third opinion, to avoid the possibility of ‘tunnel vision’.

## Discussion

### Numbers of cases

We analysed all case reports we could find where the person receiving EAS had an intellectual disability, ASD or both. The 39 identified cases are 4.1% of the total number of cases published on the RTE website. It is important to note that these numbers may not be representative of the actual numbers or percentages of people with intellectual disabilities and/or ASD who received EAS; it could be that the complex nature of many such cases makes them more likely to be selected for publication. It is also possible that we have missed some cases where the person had an intellectual disability or ASD. The case selection was based on the descriptions and terminology in the case reports and may therefore not be comprehensive. For example, an ASD diagnosis was sometimes mentioned just briefly once, so it may be that some patients with intellectual disability or ASD have not been noted within the data-set.

It is not possible, therefore, to know how typical the selected case reports are, or how common or representative the stated comorbidities and causes of suffering are within the population of people with intellectual disabilities or ASD; nor is it possible to make clear comparisons with other groups of patients (for example, those with mental health problems but without intellectual disability or ASD). However, as the RTE website states, these EAS reports have been selected for publication because of their importance in the development of societal norms and standards^10^ and to give physicians insight into the RTE's considerations.^2^ As such, they serve as guidance for physicians’ decision-making in the future. Therefore, exploring the reasons for requesting and granting these 39 EAS requests is of considerable importance.

### Are characteristics of ASD or intellectual disability acceptable reasons for EAS?

In the vast majority of granted EAS requests in The Netherlands (89%) the main stated cause of suffering is somatic.^2^ However, somatic causes accounted for only 15% of the cases in this study. In two-thirds of these cases (62%), the characteristics of ASD or intellectual disability were the sole or major contributing cause of suffering, and these were assumed to be severe enough to approve EAS.

In our study, levels of loneliness and not coping with the outside world are striking. Adverse life events, including bullying, loneliness and unemployment, are frequently experienced by this group. The acceptance of these as criteria for ending life could reflect a tacit endorsement of society’s failure of inclusion of people with ASD/intellectual disability and a failure to ensure that resources and competencies are available to assist people to cope with the challenges society and daily living present. Other researchers have noted similar reasons in EAS requests from psychiatric patients, and have argued that although these are societal factors that may be beyond the control of patients or physicians, they are not acceptable reasons for granting the request.^12^

In many cases, the suffering was described as not being able to keep up in society, feeling excluded from it, an inability to maintain relationships, depression, sadness, distress at not being the person they would like to be, and difficulty in coping with changing circumstances. These experiences are closely linked to failures in social care. For example, over a quarter of cases describe people who had difficulty coping with what they experienced as an overload of sensory stimuli, such as noise. This type of suffering in people with ASD is well recognised and sensory assessments can guide effective interventions to create autism-friendly living and working environments.^13^

Cassidy et al^14^ found that people and people with autistic traits were significantly over-represented among those who die by suicide. The cognitive inflexibility associated with ASD may reduce some people's problem-solving ability such that they cannot find a way out of a stressful situation and see suicide as the only solution.^15^

### Societal inequities and unconscious bias

The case reports contain only the physician’s account of the situation. These included some descriptions of mental capacity assessments (although limited and not analysed for this study). In assessing an EAS request, it is important that the assessor does not inadvertently reinforce the patient’s feelings of inferiority or of rejection by society. Disablism – society’s unconscious bias against physically or intellectually disabled people, often subliminally expressed – can constitute a coercive pressure on someone with a disability. For example, such attitudes were evident during the COVID-19 pandemic in the overuse of ‘do not resuscitate’ orders for people (including young people) with intellectual disability.^16^ Furthermore, people with disabilities experience stark inequities in health and social care provision, severe enough to put some patient groups at real risk^17^ and to lead to premature and avoidable deaths.^18,19^ These inequities, including societal failures to accept and provide adequate support for disabled people to manage their lives, appear to have played a not insignificant part in the EAS requests of people with intellectual disabilities and/or ASD in our study. These cases raise ethical dilemmas that society must face in defining the extent of its duty of care to its citizens, particularly those with disabilities.

Dutch law requires that EAS is permitted only in cases where the suffering has a medical basis. This raises real questions about accepting factors such as ‘difficulty in coping with changing circumstances’ as reasons for EAS, as these are associated with lifelong disability rather than an acquired medical condition. The implicit message communicated to patients in granting EAS requests on the basis of intellectual disability or ASD-related suffering is that such conditions are indeed hopeless.^20^ This is of concern, as is the risk that the option of EAS hampers investment in appropriate treatments and societal changes.

### A need for philosophical and ethical debate

We question whether applying a biomedical framework to people with complex social and psychological needs, in particular when assessing very broadly defined ‘suffering’, is overly simplistic and indeed dangerous. We believe that the issues raised in this paper warrant a much wider philosophical and ethical debate around the parameters for EAS legislation and practice. Our concerns echo those raised internationally about whether the necessarily broad criteria for legal EAS provide sufficient safeguards for vulnerable patient groups, such as those with psychiatric conditions.^21–23^ We also share concerns about the level of disagreement between physicians and the level of regulatory oversight.^24^

The cases in this study were highly complex, requiring careful consideration of the reasons behind both the EAS request and the granting of that requests. It is of crucial importance to understand how physicians assess the unbearable nature and the ‘hopelessness’ of patients’ suffering. Further investigation and discussion of such cases will contribute to the international debate on dealing with EAS requests from vulnerable patient groups.

### Strengths and limitations

The strength of this study is that, to date, this is the largest investigation of actual cases of EAS for people with intellectual disabilities or ASD. However, there are clear limitations in its reliance on the cases the RTE selected to publish, which may not be representative of all EAS cases involving people with intellectual disabilities and/or ASD. We could only assess what physicians chose to report, which was based on their own perspective. These reports were rather standardised and failed to include adequate descriptions of social circumstances or of in-depth conversations with the patient, those in their social circle and other professionals. It may well be that such descriptions were available in the patients’ medical or social care records, but by publishing these limited reports with their emphasis on describing the ‘causes of unbearable suffering’, the RTE provides implicit guidance on acceptable practice to physicians faced with similar cases in the future. We therefore believe that the questions we have raised are valid.

## Data Availability

The full case reports are publicly available (in Dutch) on this website: https://www.euthanasiecommissie.nl/. Case-number details and English-language extracts of all cases (translated by the first author), plus the authors’ categorisation, is available at the Research Data Repository of the first author's university (https://researchdata.kingston.ac.uk/168/).

## Supplementary material

For supplementary material accompanying this paper visit http://doi.org/10.1192/bjo.2023.69. click here to view supplementary material

## Appendix

### Examples illustrating the main study findings

#### Case 1 (2018–24): Suffering arising solely from having intellectual disability/ASD

A man of above-average intelligence (aged 18–30) was severely autistic and found this difficult to cope with. His suffering was described as ‘the realisation that he could not lead a “normal” life’. He had received a range of treatments and support to help him accept and manage the limitations, including psychotherapy, cognitive–behavioural therapy, electroconvulsive therapy and medication for his low mood, breathing techniques, help in structuring his days, and support in moving into a stimuli-poor independent home. Despite this, he could barely manage independently and quickly became overstimulated. In particular, he could not, or could only with great difficulty, perform daily activities that required contact with others; these then exhausted him completely. Making choices or carrying out simple instructions was extremely difficult, if not impossible, because of his rigid way of thinking, which resulted in a need for clarity, certainty and structure; this paralysed him in his functioning. Because of his inability to put himself in other people’s shoes and understand them, it was also impossible to form intimate relationships, although he wanted to. He suffered from the hopelessness of his situation and the lack of any prospect of improvement. His doctor, who had known him for 2 years, agreed that there were no realistic options left to relieve his suffering.

#### Case 2 (2020–114): Suffering arising from intellectual disability/ASD, triggered by somatic conditions

A woman in her 70s, who had mild intellectual disability and was recently diagnosed with severe ASD, had had a gastric carcinoma 18 months ago, which was successfully treated with a partial gastric resection. Her suffering consisted of not being able to cope with the adjustments in her routines that the new limitations brought with them. Owing to the partial gastric resection, she should eat small amounts several times a day, but she was unable to do this because of her rigid belief that she should always eat three times a day. She lost more and more weight, became more and more limited in her functioning and became increasingly dependent on the help of others. There was increasing loss of strength and stamina, intense fatigue, abdominal and back pain, nausea, vomiting and cachexia. The fact that the cancer had been cured made little difference to her; she experienced the profound changes in her life as impossible and unbearable. The RTE summarised her case as follows: ‘Given the severity of the patient’s ASD and the resulting need for control, order and routine, following the resection of her gastric cancer she was unable to adapt her eating patterns to the extent that would prevent malnutrition. In other words, changing her habits was more stressful for her than starvation.’

#### Case 3 (2020–11, female, 30s, ASD): Suffering arising from psychiatric conditions plus characteristics associated with intellectual disability/ASD

A woman in her 30s with ASD suffered from post-traumatic stress disorder and borderline personality disorder. She has been sexually abused in her early teens. She had received numerous treatments for her psychiatric conditions; for the past 8 years she had been treated and supported for her ASD. Despite her efforts to make the treatments work, there was no real improvement. The core of her suffering was described as an inability to love herself. She suffered from fears, limited stress tolerance, being easily overstimulated, tormenting perfectionism and an inability to live independently or maintain relationships. She was offered supported living, but her psychiatrist agreed that contacts with other residents would be too difficult owing to her ASD. The patient was tired of her struggle with life.

#### Case 4 (2019–94, male, 70s, intellectual disability): Suffering arising from somatic conditions

A man in his 70s with mild intellectual disability was diagnosed with Parkinson's disease 5 years before death. The patient's suffering consisted of difficulty in swallowing leading to pneumonias, severe stiffening that made him immobile, incontinence and painful contractures in his wrists and elbows. Up to 3 months before his death, he had lived his life as well as possible with cheerfulness and humour. In the end, his behaviour changed. He was fearful, threw things, called people names and hit people. His medication had become less effective. He deteriorated rapidly in the final year and suffered from his complete dependence and lack of hope of improving his situation.

#### Case 5 (2020–136, female, 30s, ASD): Suffering arising from psychiatric conditions

A woman in her 30s with ASD had complex comorbidities, including an anxiety disorder since early childhood, severe obsessive–compulsive disorder, a personality disorder, a psychotic disorder and Tourette syndrome. She had been detained under the Compulsory Mental Health Care Act for several years and lived on a closed in-patient ward. Her suffering consisted of continuous intrusive thoughts and compulsions. She described her life as a succession of misery, ignorance, doubt and struggle. She often felt lonely and sad. The RTE noted that it had been more difficult to treat her because of her ASD and psychotic vulnerabilities.

#### Case 6 (2020–113, female, 50s, intellectual disability): Suffering arising from a combination of somatic and psychiatric conditions

A woman in her 50s, who had mild intellectual disability, suffered from spina bifida with total paralysis of her lower body. She also had osteoarthritis, spondylosis, kyphosis, chronic sacral pressure ulcers, renal dysfunction, tinnitus, tension headache, muscle atrophy and pain in her elbows, upper arms and shoulders. She was ‘rolstoelgebonden’ (‘wheelchair-bound') and lived in a care facility. In her youth, the patient had been affectively and emotionally neglected by her foster family and she had often felt unwanted, unsafe and vulnerable. In her adult life she had to move to a different care facility 15 times; the frequent changes of support workers and caregivers had demanded a lot from her adaptability. She had always felt rejected, no longer had faith in humanity, felt overstimulated and experienced her total dependence as an unbearable burden. She increasingly suffered from the physical effects of her infirmities, especially the pain in her arms and shoulders. She had no contacts or day-to-day activities that were meaningful to her. The patient did not want to live like this anymore. Her physician thought that her disturbed neurocognitive and socio-emotional development made it too difficult for her to adapt and accept her limitations, and that the combination of somatic and psychological suffering was unbearable for her.
