# Supplementary material for: Euthanasia and physician-assisted suicide in people with intellectual disabilities and/or autism spectrum disorders: investigation of 39 Dutch case reports (2012–2021)
Source: BJPsych Open. 2023 May 23;9(3):e87. doi: 10.1192/bjo.2023.69 (PMC10228250; doi:10.1192/bjo.2023.69)
Supplement: Supplementary file 1 [file S2056472423000698sup001.zip › bjpsychopen-22-0506-20230420074417/doc/BJPsychOpen-22-0506.R3 clean.docx]

**Title**

Euthanasia and physician-assisted suicide in people with intellectual disabilities and/or autism spectrum disorders: an investigation of 39 Dutch case reports (2012-2021)

**Authors**

1. **Irene Tuffrey-Wijne** *(corresponding author)*Kingston University London
   Faculty of Health, Science, Social Care and Education
   Kingston Upon Thames KT2 7LB
   UK
   *Email:* I.Tuffrey-Wijne@kingston.ac.uk
2. **Leopold Curfs**Governor Kremers Centre, Maastricht University Medical Centre, Netherlands
3. **Sheila Hollins**Kingston University London, Kingston Upon Thames, UK
4. **Ilora Finlay**Cardiff University, Wales, UK

**Introduction**

This paper reports on an analysis of 39 cases in the Netherlands (2012-2021) where physicians performed euthanasia or assisted the suicide of people who had an intellectual disability, autism spectrum disorder, or both.

**Euthanasia and assisted suicide (EAS) in the Netherlands**

The Netherlands’ 2002 *Termination of Life on Request and Assisted Suicide Act* made it legally possible to perform euthanasia (where a physician administers a fatal dose of a drug to the patient at his or her express request) or physician-assisted suicide (where the physician supplies the drug, but the patient administers it), provided six due care criteria are met (see Supplementary File 1). Physicians carrying out the EAS must report each case; these reports are examined by a euthanasia review committee *(Regionale Toetsingscommissie Euthanasie* (RTE)), tasked with adjudicating whether the requirements of “due care” had been observed and met. One of the due care criteria, and the focus of this paper, is that “the patient’s suffering is unbearable, with no prospect of improvement”. In their EAS report, physicians must explain what the suffering consisted of, why they were convinced it was unbearable, and how they came to the conclusion that there was no prospect of improvement.^1^

In 2021 in the Netherlands, EAS accounted for 7,666 deaths (4.5% of all deaths).^2^ The vast majority of granted EAS requests (89%) were for patients whose “unbearable suffering” was caused by somatic conditions, predominantly cancer. However, a life-limiting condition is not a prerequisite for granting an EAS request. Dutch law requires the “unbearable suffering” to have a medical basis, but this can be either somatic or psychiatric. It permits EAS for unbearable suffering caused by psychiatric conditions, dementia, multiple geriatric syndromes, chronic pain syndromes, or genetic conditions. Whilst the percentage of these EAS requests may be low, the numbers of deaths through EAS for reasons other than terminal illness are increasing and not insignificant.

**Intellectual Disability (ID) and Autism Spectrum Disorder (ASD)**

People fall within the definition of ID if all of the following apply: (1) limitations (“deficits”) in intellectual functioning (IQ score ≤70); (2) impairments in adaptive and/or social functioning; (3) these limitations occur before adulthood and are lifelong. ASD is a complex and usually lifelong developmental disorder, characterised by persistent difficulties in social communication and social interaction across multiple contexts. This can include deficits in social-emotional reciprocity, in nonverbal communicative behaviours, and in developing, maintaining and understanding relationships. Although ASD is common among people with ID, not all people with ASD have an ID; they may have average or above-average intelligence.^3^

Estimates of prevalence are around 1-2% of the population for ID^4,5^ and 1-2% for ASD.^6^

**Euthanasia and physician-assisted suicide in patients with ID and/or ASD**

People with ID or ASD are not excluded from making, and being granted, an EAS request. Their right to do so is in line with the UN Convention on the Rights of Persons with Disabilities.^7^ The significant complexities and ethical dilemmas in such cases were highlighted in an examination of nine cases of EAS granted to people with ID and/or ASD; only two of these had progressive and life-limiting somatic conditions. Assessment of suffering was particularly difficult for patients with life-long disability.^8^

**Aims**

The aims of this study are to (1) describe the characteristics and circumstances of people with ID or ASD who were granted their EAS request; (2) investigate the main causes of suffering and the factors associated with or contributing to the experience of unbearable suffering which led to the EAS request; and (3) examine how physicians assessed and responded to the EAS request.

This paper follows the Standards for Reporting Qualitative Research (SRQR) reporting guideline.

**Methods**

**Researcher characteristics**

The authors of this paper are experts in the fields of intellectual disability and palliative care. We come from the Netherlands and the UK, which have divergent legal frameworks with regards to EAS. Our aim is not to present or promote a common ethical perspective, but to contribute to the international debate by presenting the findings of a rigorously conducted study of public data and to present our assessment of the implications. The first and second authors are native Dutch speakers and fluent English speakers.

**Sample**

Between 2012 and 2021, the Dutch RTEs received 59,996 notifications of EAS; case summaries of 927 of these (1.5%) are included (in Dutch) in a searchable open access database on the RTE website, with the specific aim to show how the committee applied and interpreted the legal due care criteria, and how they dealt with particular challenges.^9^ No pre-2012 case reports are available.

We searched for case reports involving people who had ID and/or ASD by using the following Dutch keywords: *verstandelijk, verstandelijke beperking, intellectuele beperking, zwakbegaafd, verminderde intelligentie, autisme, ASS, Asperger.* Case reports where the person did not have ID or ASD (for example, they had cognitive limitations due to dementia, or they had been assessed for ASD but found not to have it) were excluded from the results. This left a total of 39 relevant cases for inclusion in the study; reportedly 15 people had ID, 20 patients had ASD, and 4 people had both ID and ASD (see Supplementary File 2).

The 39 case reports were between 852 words and 4,436 words long (median 1,907 words), describing the physician’s written reports of the nature of the patient’s suffering, possible alternatives to EAS, discussions between physicians and patient, the EAS request, the consultations with other physicians (including second independent opinion), how the EAS was carried out, any further verbal explanations requested from the physician by the RTE, and the considerations and verdict of the RTE.

**Data analysis**

We performed inductive thematic analysis of the data (Thomas 2006), using the Framework Method^10^ supported by Nvivo software (v.12). The steps were as follows: (1) *Familiarisation:* The first author read and re-read all 39 case reports; the second author (LC) read a selection of case reports. The first author translated a summary of each case into English, which was read by all four authors. (2) *Initial inductive coding:* reading the cases line by line, the first author applied a label (“code”) to all possible factors and contributors, resulting in a total of 353 coded segments. (3) *Developing a coding framework:* the first and second authors discussed the codes and developed a detailed coding framework consisting of 22 categories. The framework was discussed and agreed with all four authors. (4) *Final coding:* the first author coded all 39 full documents into the agreed framework. This allowed for frequency tabulation as well as descriptive overviews of the contributing factors. (5) *Final categorisation:* The main causes of suffering were categorised according to whether they related to characteristics of ASD/ID, somatic conditions, psychiatric conditions, or a combination of these. Each case summary was re-read and discussed with all four authors to reach consensus on final categorisation.

**Results**

**Patient characteristics and circumstances**

An overview of patient characteristics and circumstances is given in Table 1.

*-----------------------------------Table 1 about here--------------------------------------------------------------------*

*Characteristics*

Nineteen patients (49%) had ID and 24 patients (62%) had ASD; of these, 4 had both ID and ASD (10%). All age groups were represented, with 18 patients (46%) younger than 50 when they died. A very wide range of diagnosed somatic conditions were mentioned for 26 patients (67%), with most having more than one condition, but no single somatic condition predominant. Cancer (the predominant cause of suffering in 61% of all EAS cases in the Netherlands) was mentioned for 3 patients (8%). Psychiatric conditions were mentioned for 25 patients (64%). Of these, depression was the most common (31% of patients), followed by obsessive compulsive disorder (OCD), borderline personality disorder and psychosis (26%, 21% and 21% respectively).

Other notable characteristics included suicidal thoughts or suicide attempts (44%), childhood trauma including abuse and neglect (18%), adult trauma including life events such as bereavements (21%) and substance abuse (13%).

*Circumstances*

Descriptions of the patients’ social circumstances were limited. Whilst past psychiatric in-patient episodes (in some cases frequent) were mentioned in 41% of cases, current living situations or social support structures were not always made clear, apart from 8 patients (21%) who were in supported living or residential settings. More than two thirds of case reports made no mention of the patient’s family or other significant people in their lives. Of the 12 case reports (31%) where family was mentioned, only half (15%) indicated that EAS discussions had involved family, or that family was present at the death. The others mentioned the existence of family just briefly, such as:

“The patient was unable to make friends and had become isolated, including within her own family.” *(2017-80, female, age 18-30, ASD)*

**Main causes of suffering**

The main cause of suffering that led to the EAS request are summarised in Table 2. Full case examples are given in Table 3, to illustrate the different factors contributing to the patients’ experience of unbearable suffering.

*-----------------------------------Tables 2 and 3 about here----------------------------------------------------------------*

In 8 cases (21%), the only causes of suffering described were factors directly associated with ID or ASD. Typically, these people were unable to live with the characteristics of ASD/ID and could not cope with the world:

“As he had never been able to keep up with society, he had become insecure with recurring depression. Due to his intellectual disability, he felt a great pressure of the world on him which he could not handle. His autistic traits made it increasingly difficult for him to cope with changes around him.” *2020-27, male, 70s, ID & ASD*

In 8 cases (21%), ASD or ID made it difficult to cope with non-life-threatening somatic symptoms or physical decline, such as age-related conditions or symptoms (n=5), tinnitus (n=2) or curable cancer (n=1).

“The routine that she had introduced into her life also gave the patient something to hold on to when dealing with a world that was complex for her… The progressive physical deterioration and the lifelong inability to deal with her environment other than in fixed patterns, caused unbearable suffering.” *2018-14, female, 80s, ASD*

In a further 8 cases (21%), ASD/ID was a major contributing factor to the person’s inability to cope with their psychiatric condition; or the main causes of suffering were described as a combination of psychiatric conditions and the characteristics associated with ASD or ID. In one case, there was an additional somatic cause of suffering (chronic fatigue syndrome).

“The patient mainly suffered from anxiety, compulsive complaints and loneliness due to the limitations that arose from ASD, obsessive compulsive disorder, acquired brain injury, and personality disorder.” *2020-150, male, 40s, ASD*

In 15 cases (38%), the person’s EAS request stemmed from suffering that was not substantially related to their ASD or ID, but related to psychiatric conditions (n=6), somatic conditions (n=6; all of these were people with ID) or a combination (n=3).

**Factors associated with the experience of unbearable suffering**

Table 4 gives a summary of the factors associated with or contributing to the experience of unbearable suffering which led to the EAS request. The following factors were particularly associated with having ASD and/or ID: social isolation and loneliness, a lack of coping or resilience, lack of flexibility, and oversensitivity to stimuli.

*-----------------------------------Table 4 about here--------------------------------------------------------------------*

*Social isolation and loneliness*

Over three quarters of patients described being lonely or socially isolated as a major cause of suffering. This often stemmed from feeling rejected and different from others. For patients with ASD in particular, their difficulty in making or coping with social contacts was a major factor.

“The patient had felt unhappy since childhood and was persistently bullied because he was just a bit different from others… [He] longed for social contacts but was unable to connect with others. This reinforced his sense of loneliness. The consequences of his autism were unbearable for him... The prospect of having to live on in this way for years was an abomination to him and he could not muster it.” *(2021-26, Male, 20s, ASD)*

“The patient suffered from his inability to participate in society… [He] was not able to live among people, because he was easily over-stimulated. This made him isolated.” *(2019-22, male, 70s, ASD)*

“She suffered from the social isolation that her behaviour had led to. Meetings were disturbed by her shouting. People thought her repulsive and nobody wanted to be near her. She was unable to give her life meaning in any other way.” *(2018-12, female, 80s, ID)*

*Lack of resilience or coping*

For more than half of patients (n=22, 56%), difficulty in coping with life or with the world (often described as a lack of resilience) was a major contributor to their EAS request. For some, this had been a life-long cause of unbearable suffering:

“Panic and despair were constant companions. The patient felt powerless to function in today’s society and could not be the person he wanted to be, with a job and a family.” *(2018-69, male, 50s, ID and ASD)*

“The patient found the world too complex.” *(2019-22, male, 70s, ASD)*

For others, somatic or psychiatric deterioration tipped them over the edge, as their coping ability was exceeded:

“The patient had insufficient strategies to cope with his illness.” *(2018-27, male, 70s, ID)*

“The patient could not cope with losing her fixed routines and with her increased dependence. This made her frustrated, desperate and sad.” *(2018-14, 80s, ASD)*

*Lack of flexibility*

Rigid coping strategies, a need to stick to routines, difficulties in considering alternatives, and compulsive behaviours were a major cause of suffering for 17 patients (44%). For them, this lack of flexibility tipped the balance and was a major factor in their EAS request.

“With her primitive mind, the patient was only focused on the complete removal of the tinnitus. The moment until her realized “I will never get rid of it”, her suffering had become hopeless and unbearable for her, and she was only focused on euthanasia.” *(2015-83, female, 60s, ID)*

“She rejected help from others because she wanted to keep doing everything herself, following fixed rituals, even when that was barely possible anymore.” *(2016-48, female, 90s, ID & ASD)*

“The independent psychiatrist was of the opinion that the severity of suffering was related to his limited coping mechanisms and flexibility, stemming from ASD, with a fixation on the problem rather than an ability to let go and bear it.” *(2020-33, male, 50s, ASD)*

*Oversensitive to stimuli*

Of the 10 patients for whom an oversensitivity to stimuli was a cause of suffering leading to their EAS request, nine had ASD. Sensitivity to stimuli was usually described as part of a list of other difficulties. For example, one patient was described as suffering from fits of anger caused by fear, obsessively holding on to routines, overwhelming sadness and unexplained, intractable pain:

“She also suffered from over-sensitivity to stimuli such as noises, temperature or touch... she was barely resilient due to being easily over-stimulated.” *(2017-80, female, age 18-30, ASD)*

**Physician and RTE response**

Physicians had the task of ascertaining that the patients’ suffering was unbearable and had no prospect of improvement. This was not easy. Most cases were highly complex, with multiple factors contributing to the EAS request.

The request was agreed, and EAS carried out, by the patient’s own physician (usually the GP) in 7 cases (18%) and a psychiatrist or mental health services in 5 cases (13%). The majority of cases (27, 69%) was assessed and agreed by the Expertise Centre Euthanasia (ECE), mostly (n=22) because the patient’s own physician found the case too complex. (The ECE consists of teams of physicians and nurses who offer EAS to patients whose request was denied by their own physician. If they find that all due care criteria are met, an ECE physician can carry out the EAS.^11^)

*Assessment of unbearable suffering*

In a third of cases, physicians noted explicitly that ASD and ID are not treatable, and that this was the key consideration in their assessment that there was no prospect of improvement for the patient’s suffering.

“ASD is incurable and its treatment is purely symptomatic.” *(2019-99, female, 30s, ASD)*

“ASD can be supported, but it can’t be treated.” *(2020-113, female, 50s, ASD & ID)*

Physicians noted that fundamentally, the patient’s suffering stemmed from the limitations of ASD or ID:

“Because of this, the patient was unable to build a ‘normal’ life… The physician thought there was no prospect of improvement. Learning to live with his limitation would be the only option for the patient.” *(2020-44, male, 40s, ASD)*

“According to [the physician], the huge burden of suffering had to be seen in the context of the patient’s limited resilience and coping, which was a result of his limited intelligence and near-absence of the ability to reflect.” *(2018-27, male, 70s, ID)*

The fact that treatments hadn’t helped, and wouldn’t help, was noted by physicians in 31 cases (79%); this was often strongly influenced by the perceived limitations of ASD or ID, which led to a lack of flexibility, adaptability, and resilience:

“The patient’s personality and her intellectual limitations resulted in an inability to profit from psychological or psychiatric interventions.” *(2015-24, female, 60s, ID)*

“His intellectual disability and affective neglect in childhood had led to insufficient resilience to cope with suffering.” *(2018-71, male, 50s, ID)*

Where the suffering consisted solely or significantly of the way ASD or ID affected the patient, physicians sometimes struggled to apply the EAS due care criteria. In 14 cases (36%) the assessing physician did not think that the criteria for EAS were met.

“According to [the first consultant], because there was no medical basis for the suffering, not all requirements had been met.” *(2018-27, male, 70s, ID)*

In most of those cases, the patient was referred to the ECE for further assessment, and/or seen by another consultant, eventually leading to the conclusion that EAS was the right option for the patient.

**RTE verdict**

In two cases (2017-14 and 2018-69) the RTE concluded that the due care criteria had not been met, because of the physician’s failure to seek adequate advice from independent consultants. In case 2018-69 (male, 50s, ASD and ID) the independent consultant who assessed the patient concluded that, even though the main diagnosis (ASD) was untreatable, there were still options for improving his resilience and for helping him cope better with the death of a parent; therefore, the legal EAS criteria had not been met. The patient’s physician disagreed and carried out the euthanasia. The RTE’s verdict was that the physician should have sought a third opinion, to avoid the possibility of ‘tunnel vision’.

**Discussion**

We analysed all case reports we could find where the person receiving EAS had ID, ASD or both. The 39 identified cases are 4.1% of the total number of cases published on the RTE website. It is important to note that these numbers may not be representative of the actual numbers or percentages of people with ID and/or ASD who received EAS; it could be that the complex nature of many such cases makes them more likely to be selected for publication. It is also possible that we have missed some cases where the person had ID or ASD. The case selection was based on the descriptions and terminology in the case reports and may therefore not be comprehensive. For example, an ASD diagnosis was sometimes mentioned just briefly once, so it may be that some patients with ID or ASD have not been noted within the dataset.

It is not possible, therefore, to know how typical the selected case reports are, or how common or representative the stated co-morbidities and causes of suffering are within the population of people with ID or ASD; nor is it possible to make clear comparisons with other groups of patients (for example, those with mental health problems but without ID or ASD). However, as the RTE states, these EAS reports have been selected for publication because of their importance in the development of societal norms and standards^9^ and to give physicians insight into the RTE’s considerations^2^. As such, they serve as guidance for physicians’ decision-making in the future. Therefore, exploring the reasons for requesting and granting these 39 EAS requests is of considerable importance.

In the vast majority of granted euthanasia requests in the Netherlands (89%) the main stated cause of suffering is somatic. However, somatic causes accounted for only 15% of the cases in this study. In two thirds of cases (62%), the characteristics of ASD or ID were the sole or major contributing cause of suffering, and these were assumed to be severe enough to approve EAS.

In our study, levels of loneliness and not coping with the outside world are striking. Adverse life events including bullying, loneliness, and unemployment are frequently experienced by this group. The acceptance of these as criteria for ending life could reflect a tacit endorsement of society’s failure of inclusion of people with ASD/ID, and a failure to ensure the resources and competencies are available to assist people to cope with the challenges society and daily living present. Other researchers have noted similar reasons in EAS requests from psychiatric patients, and have argued that whilst these are societal factors that may be beyond the control of patients or physicians, they are not acceptable reasons for granting the request.^12^

In many cases, the suffering was described as not being able to keep up in society, feeling excluded from it, an inability to maintain relationships, depression, sadness, distress at not being the person they would like to be, and difficulty in coping with changing circumstances. These experiences are closely linked to failures in social care. For example, over a quarter of cases describe people who had difficulty coping with what they experienced as an overload of sensory stimuli, such as noise. This type of suffering in people with ASD is well recognised and sensory assessments can guide effective interventions to create autism-friendly living and working environments.^13^

Cassidy et al^14^ found that autistic people and people with autistic traits were significantly over-represented amongst those who die by suicide. The cognitive inflexibility associated with ASD may reduce some people’s problem-solving ability such that they cannot find a way out of a stressful situation and see suicide as the only solution.^15^

The case reports only contain the physician’s account of the situation. These included some descriptions of mental capacity assessments (although limited and not analysed for this study). In assessing any such request, it is important that the assessor did not inadvertently reinforce the patient’s feelings of inferiority or of rejection by society. Disablism – society’s unconscious bias towards physically or intellectually disabled people, often subliminally expressed, can constitute a coercive pressure on the disabled person. For example, such attitudes were evident during the COVID-19 pandemic in the overuse of Do Not Resuscitate orders for people (including young people) with ID.^16^ Furthermore, people with disabilities experience stark inequities in health and social care provision, severe enough to contribute to put some patient groups at real risk^17^ and to lead to premature and avoidable deaths.^18,19^ These inequities, including societal failures to accept and provide adequate support for disabled people to manage their lives, appeared to have played a not insignificant part in the EAS requests of people with ID and/or ASD. These cases raise ethical dilemmas that society must face in defining the extent of its duty of care to its citizens, particularly those with disabilities.

Dutch law requires that EAS is only permitted in cases where the suffering has a medical basis. This raises real questions about accepting factors such as ‘difficulty in coping with changing circumstances’ as reasons for EAS, as these are associated with life-long disability rather than an acquired medical condition. The implicit message communicated to patients in granting EAS requests on the basis of ID or ASD-related suffering is that such conditions are indeed hopeless.^20^ This is of concern, as is the risk that the option of EAS hampers investments in appropriate treatments and societal changes.

We question whether applying a biomedical framework to people with complex social and psychological needs, in particular when assessing very broadly defined ‘suffering’, is overly simplistic and indeed dangerous. We believe that the issues raised in this paper warrant a much wider philosophical and ethical debate around the parameters for EAS legislation and practice. Our concerns echo those raised internationally around the question whether the necessarily broad criteria for legal EAS provide sufficient safeguards for vulnerable patient groups, such as those with psychiatric conditions.^21–23^ We also share concerns about the level of disagreement between physicians and the level of regulatory oversight.^24^

These cases were highly complex, requiring careful consideration of the reasons behind both the EAS request and the granting of that requests. It is of crucial importance to understand how physicians assess the unbearable nature and the ‘hopelessness’ of patients’ suffering. Further investigation and discussion of such cases will contribute to the international debate on dealing with EAS requests from vulnerable patient groups.

**Strengths and limitations**

The strength of this study is that to date, this is the largest investigation of actual cases of EAS for people with ID or ASD. However, there are clear limitations in its reliance on the cases the RTE selected to publish, which may not be representative of all EAS cases involving people with ID and/or ASD. We could only assess what physicians chose to report, which was based on their own perspective. These reports were rather standardized and failed to include adequate descriptions of social circumstances or of in-depth conversations with the patient, those in their social circle, and other professionals. It may well be that such descriptions were available in the patients’ medical or social care records, but by publishing these limited reports with their emphasis on describing the ‘causes of unbearable suffering’, the RTE provides implicit guidance on acceptable practice to physicians faced with similar cases in the future. We therefore believe that the questions we have raised are valid.

**Declaration of interest:** None

**Funding:** None

**Author contributions:** All authors contributed to the concept and design of this study. Data extraction and detailed data analysis: 1^st^ author, with contributions from 2^nd^ author. Overal data analysis: all authors. The 1^st^ author drafted the manuscripts, which was discussed and agreed with all authors.

**Data availability:** The full case reports are publicly available (in Dutch) on this website: https://www.euthanasiecommissie.nl/
Case number details and English-language extracts of all cases (translated by first author), plus the authors’ categorisation, are available on the Research Data Repository of the first author’s university.

**Ethics statement:** Both the Kingston University Research Ethics Committee (London, UK) and the Legal Council of University Hospital Maastricht (Netherlands) confirmed that our study was exempt from ethical review, so no informed consent was required. We used anonymized data that are publicly available on the RTE website. The content of the RTE website is subject to a Creative Commons Zero (CC0) declaration, which means that re-use is permitted *(https://www.euthanasiecommissie.nl/copyright).*^9^ The RTE states that they select anonymized cases for publication, based on their relevance to the development of standards and their importance in terms of public and societal interest.^9^ We discussed our study with a lawyer who is responsible for ethical issues involved in the use of the RTE database. They highlighted that a committee appointed by the Dutch government, which oversees ethical issues around use of the database, has recommended that the published data may be used on commented on freely and without need for further ethical approvals, as long as they are not combined with any other data sets (such as death records) that might compromise anonymity. Our study protocol involved scrutiny of the publicly available data only, and therefore, the lawyer confirmed that as these have already been subject to Dutch government considerations, further ethical approvals were not required.

**References**

1. Regional Euthanasia Review Committees. *Euthanasia Code 2018*.; 2019. Accessed December 17, 2022. https://www.euthanasiecommissie.nl/euthanasiecode-2018/uitspraken/brochures/brochures/euthanasiecode/2018/euthanasia-code-2018

2. RTE (Regional Euthanasia Review Committees). *Regional Euthanasia Review Committees Annual Report 2021*.; 2022. Accessed December 17, 2022. https://www.euthanasiecommissie.nl/de-toetsingscommissies/jaarverslagen

3. American Psychiatric Association. *Diagnostic and Statistical Manual of Mental Disorders: DSM-5*. 5th ed. American Psychiatric Publishing; 2013.

4. Learning Disabilities Observatory. *People with Learning Disabilities in England 2015 : Main Report*. Public Health England; 2016. https://www.improvinghealthandlives.org.uk/securefiles/161129_1604//PWLDIE 2015 final.pdf

5. Maulik PK, Mascarenhas MN, Mathers CD, Dua T, Saxena S. Prevalence of intellectual disability: a meta-analysis of population-based studies. *Res Dev Disabil*. 2011;32(2):419-436. doi:10.1016/j.ridd.2010.12.018

6. Chiarotti F, Venerosi A. Epidemiology of autism spectrum disorders: A review of worldwide prevalence estimates since 2014. *Brain Sci*. 2020;10(5). doi:10.3390/brainsci10050274

7. United Nations. Convention on the rights of persons with disabilities. Published 2006. Accessed November 20, 2017. https://www.un.org/development/desa/disabilities/convention-on-the-rights-of-persons-with-disabilities/convention-on-the-rights-of-persons-with-disabilities-2.html

8. Tuffrey-Wijne I, Curfs L, Finlay I, Hollins S. Euthanasia and assisted suicide for people with an intellectual disability and/or autism spectrum disorder: an examination of nine relevant euthanasia cases in the Netherlands (2012 – 2016). *BMC Med Ethics*. 2018;19(17):1-21.

9. RTE. Regionale Toetsingscommissies Euthanasie. Accessed December 17, 2022. https://www.euthanasiecommissie.nl/

10. Gale N, Heath G, Cameron E, Rashid S, Redwood S. Using the framework method for the analysis of qualitative data in multi-disciplinary health research. *BMC Med Res Methodol*. 2013;13:117.

11. Expertisecentrum Euthanasie. Expertisecentrum Euthanasie. Published 2019. Accessed December 17, 2022. https://expertisecentrumeuthanasie.nl/

12. Verhofstadt M, Thienpont L, Peters GJY. When unbearable suffering incites psychiatric patients to request euthanasia: qualitative study. *Br J Psychiatry*. 2017;211(4):238-245. doi:10.1192/bjp.bp.117.199331

13. Quin A, Wood A, Lodge K, Hollins S. Listening to the experts - person centred approaches to supporting autistic people and people with learning disabilities in the mental health system. *British Journal of Psychiatry Advances* (accepted for publication)

14. Cassidy S, Au-Yeung S, Robertson A, et al. Autism and autistic traits in those who died by suicide in England. *British Journal of Psychiatry*. 2022;221(5):683-691. doi:10.1192/bjp.2022.21

15. O’Connor RC, Kirtley OJ. The integrated motivational-volitional model of suicidal behaviour. *Philosophical Transactions of the Royal Society B: Biological Sciences*. 2018;373(1754). doi:10.1098/rstb.2017.0268

16. Care Quality Commission. *Protect, Respect, Connect - Decisions about Living and Dying Well during COVID-19: CQC’s Review of ‘Do Not Attempt Cardiopulmonary Resuscitation’ Decisions during the COVID-19 Pandemic*.; 2021. Accessed December 15, 2022. https://www.cqc.org.uk/publications/themed-work/protect-respect-connect-decisions-about-living-dying-well-during-covid-19

17. Bhui K, Malhi GS. Proposed Assisted Dying Bill: implications for mental healthcare and psychiatrists. *British Journal of Psychiatry*. 2022;221(1):374-376. doi:10.1192/bjp.2022.41

18. Heslop P, Blair P, Fleming P, Hoghton M, Marriott A, Russ L. *Confidential Inquiry into Premature Deaths of People with Learning Disabilities (CIPOLD)*.; 2013. Accessed January 6, 2023. http://www.bris.ac.uk/cipold/reports/index.html

19. White A, Sheehan R, Ding J, et al. *LeDeR Annual Report 2021: Learning from Lives and Deaths - People with a Learning Disability and Autistic People*.; 2022. Accessed December 1, 2022. https://leder.nhs.uk/resources/annual-reports

20. Appelbaum P. Physician-assisted death in psychiatry. *World Psychiatry*. 2018;17(2):145-146. doi:10.1002/wps.20546

21. Kim S, Lemmens T. Should assisted dying for psychiatric disorders be legalized in Canada? *Can Med Assoc J*. 2016;188(14):E337-E339. doi: [10.1503/cmaj.160365](https://doi.org/10.1503%2Fcmaj.160365)

22. Grassi L, Folesani F, Marella M, et al. Debating Euthanasia and Physician-Assisted Death in People with Psychiatric Disorders. *Curr Psychiatry Rep*. 2022;24(6):325-335. doi:10.1007/s11920-022-01339-y

23. Nicolini ME, Nicolini ME, Kim SYH, Churchill ME, Gastmans C. Should euthanasia and assisted suicide for psychiatric disorders be permitted? A systematic review of reasons. *Psychol Med*. 2020;50(8):1241-1256. doi:10.1017/S0033291720001543

24. Kim SYH, de Vries R, Peteet JR. Euthanasia and assisted suicide of patients with psychiatric disorders in the Netherlands 2011 to 2014. *JAMA Psychiatry*. 2016;73(4):362-368. doi:10.1001/jamapsychiatry.2015.2887
