# Supplementary material for: Euthanasia and physician-assisted suicide in people with intellectual disabilities and/or autism spectrum disorders: investigation of 39 Dutch case reports (2012–2021)
Source: BJPsych Open. 2023 May 23;9(3):e87. doi: 10.1192/bjo.2023.69 (PMC10228250; doi:10.1192/bjo.2023.69)
Supplement: Supplementary file 1 [file S2056472423000698sup001.zip › bjpsychopen-22-0506-20230420074417/graphic/EAS-ID-ASD_Table 1.docx]

**Table 1: Patient characteristics and circumstances (N = 39)**

| **Characteristic** | **Number of cases** | **% of cases** |
| --- | --- | --- |
| **ID or ASD** |  |  |
| ID only | 19 | 49% |
| ASD only | 24 | 62% |
| Both ID and ASD | 4 | 10% |
| **Gender** |  |  |
| Male | 19 | 49% |
| Female | 20 | 51% |
| **Age** |  |  |
| 18-29 | 5 | 13% |
| 30-39 | 7 | 18% |
| 40-49 | 6 | 15% |
| 50-59 | 5 | 13% |
| 60-69 | 4 | 10% |
| 70-79 | 7 | 18% |
| 80-89 | 3 | 8% |
| 90+ | 2 | 5% |
| **Somatic conditions** | **26** | **67%** |
| Arthritis/Osteoporosis | 7 | 18% |
| Cancer | 3 | 8% |
| Chronic Fatigue Syndrome | 1 | 3% |
| Diabetes | 3 | 8% |
| Epilepsy | 2 | 5% |
| Fractures | 2 | 5% |
| Gallstones | 1 | 3% |
| Heart & circulation | 5 | 13% |
| Kidney & bladder | 2 | 5% |
| Lungs | 5 | 13% |
| Multiple Geriatric Syndromes | 3 | 8% |
| Paralysis | 1 | 3% |
| Parkinson’s Disease | 2 | 5% |
| Sensory (hearing, sight) | 7 | 18% |
| Medically Unexplained Physical Symptoms (MUPS) | 4 | 10% |
| Spina Bifida | 1 | 3% |
| Tinnitus | 2 | 5% |
| Tourette’s Syndrome | 1 | 3% |
| **Psychiatric conditions** | **25** | **64%** |
| ADHD | 1 | 3% |
| Bipolar Disorder | 1 | 3% |
| Borderline Personality Disorder | 8 | 21% |
| Depression | 12 | 31% |
| Dissociative Disorder | 1 | 3% |
| Obsessive Compulsive Disorder (OCD) | 10 | 26% |
| Paranoia | 3 | 8% |
| Personality Disorder | 6 | 15% |
| Psychosis | 8 | 21% |
| Psychosocial problems | 3 | 8% |
| Post-Traumatic Stress Disorder (PTSD) | 4 | 10% |
| Pyromania | 1 | 3% |
| Somatoform Disorder | 2 | 5% |
| **Other characteristics** |  |  |
| Suicidal | 17 | 44% |
| Trauma (childhood) | 7 | 18% |
| Trauma (adult) | 8 | 21% |
| Substance abuse | 5 | 13% |
| **Social circumstances** |  |  |
| Supported living / residential care | 8 | 21% |
| Previous psychiatric in-patient episodes | 16 | 41% |
| Family mentioned in report | 12 | 31% |
| Family involved in EAS process | 6 | 15% |
