# Supplementary material for: Euthanasia and physician-assisted suicide in people with intellectual disabilities and/or autism spectrum disorders: investigation of 39 Dutch case reports (2012–2021)
Source: BJPsych Open. 2023 May 23;9(3):e87. doi: 10.1192/bjo.2023.69 (PMC10228250; doi:10.1192/bjo.2023.69)
Supplement: Supplementary file 1 [file S2056472423000698sup001.zip › bjpsychopen-22-0506-20230420074417/graphic/EAS-ID-ASD_Table 2.docx]

**Table 2: Main cause of suffering (N = 39)**

| **Main cause of suffering**  Factors associated with: | **Number of cases**  **(ID/ASD/ID&ASD)** | **% of cases** | **Case Example (see Table 3)** |
| --- | --- | --- | --- |
| 1. ID/ASD only | 8 (1/6/1) | 21% | 2018-24 |
| 1. ID/ASD, triggered by somatic conditions | 8 (1/5/2) | 21% | 2020-114 |
| 1. Combination of ID/ASD & psychiatric conditions | 8 (1/6/1) | 21% | 2020-11 |
| 1. Somatic conditions | 6 (6/0/0) | 15% | 2019-94 |
| 1. Psychiatric conditions | 6 (3/3/0) | 15% | 2020-136 |
| 1. Combination of somatic & psychiatric conditions | 3 (3/0/0) | 8% | 2020-113 |
