# Supplementary material for: Euthanasia and physician-assisted suicide in people with intellectual disabilities and/or autism spectrum disorders: investigation of 39 Dutch case reports (2012–2021)
Source: BJPsych Open. 2023 May 23;9(3):e87. doi: 10.1192/bjo.2023.69 (PMC10228250; doi:10.1192/bjo.2023.69)
Supplement: Supplementary file 1 [file S2056472423000698sup001.zip › bjpsychopen-22-0506-20230420074417/graphic/EAS-ID-ASD_Table 3.docx]

**Table 3: Examples illustrating the main study findings**

***Case 1*** *(2018-24):* ***Suffering arising solely from having ID/ASD***

A man of above-average intelligence (aged 18-30) was severely autistic and found this difficult to cope with. His suffering was described as “the realisation that he could not lead a 'normal' life”. He had received a range of treatments and support to help him accept and manage the limitations, including psychotherapy, cognitive behavioural therapy, ECT and medication for his low mood, breathing techniques, help in structuring his days, and support in moving into a stimuli-poor independent home. Despite this, he could barely manage independently and quickly became overstimulated. In particular, he could not, or only with great difficulty, perform daily activities that required contact with others; these then exhaust him completely. Making choices or carrying out simple instructions was extremely difficult, if not impossible, because of his rigid way of thinking and resulting need for clarity, certainty, and structure; this paralyzed him in his functioning. Because of his inability to put himself in other people’s shoes and understand them, it was also impossible to form intimate relationships, although he wanted to. He suffered from the hopelessness of his situation and the lack of any future perspective. His doctor, who had known him for two years, agreed that there were no realistic options left to relieve his suffering.

***Case 2*** *(2020-114):* ***Suffering arising from ID/ASD, triggered by somatic conditions***

A woman in her 70s, who had mild ID and was recently diagnosed with severe ASD, had a gastric carcinoma a year and a half ago, which was successfully treated with a partial gastric resection. Her suffering consisted of not being able to cope with the adjustments in her routines that the new limitations brought with them. Due to the partial gastric resection, she should eat small amounts several times a day, but she was unable to do this because of her rigid belief that she should always eat three times a day. She lost more and more weight, became more and more limited in her functioning, and became increasingly dependent on the help of others. There was increasing loss of strength and stamina, intense fatigue, abdominal and back pain, nausea, vomiting and cachexia. The fact that the cancer had been cured made little difference to her; she experienced the profound changes in her life as impossible and unbearable. The RTE summarised her case as follows: “Given the severity of the patient’s ASD and the resulting need for control, order and routine, following the resection of her gastric cancer she was unable to adapt her eating patterns to the extent that would prevent malnutrition. In other words, changing her habits was more stressful for her than starvation.”

***Case 3*** *(2020-11, female, 30s, ASD):* ***Suffering arising from psychiatric conditions plus characteristics associated with ID/ASD***

A woman in her 30s with ASD suffered from post-traumatic stress disorder (PTSD) and borderline personality disorder. She has been sexually abused in her early teens. She had received numerous treatments for her psychiatric conditions; for the past eight years she had been treated and supported for her ASD. Despite her efforts to make the treatments work, there was no structural improvement. The core of her suffering was described as an inability to love herself. She suffered from fears, limited stress-tolerance, being easily over-stimulated, tormenting perfectionism, and an inability to live independently or maintain relationships. She was offered supported living, but her psychiatrist agreed that contacts with other residents would be too difficult due to her ASD. The patient was tired of her struggle with life.

***Case 4*** *(2019-94, male, 70s, ID):* ***Suffering arising from somatic conditions***

A man in his 70s with mild intellectual disabilities was diagnosed with Parkinson's disease five years before death. The patient's suffering consisted of difficulty in swallowing leading to pneumonias, severe stiffening that made him immobile, incontinence and painful contractures in his wrists and elbows. Up to three months before his death, he had lived his life as well as possible with cheerfulness and humour. In the end, his behaviour changed. He was fearful, threw things, called names and hit people. His medication had become less effective. He deteriorated rapidly in the final year and suffered from his complete dependence and lack of perspective on improving his situation.

***Case 5*** *(2020-136, female, 30s, ASD):* ***Suffering arising from psychiatric conditions***

A woman in her 30s with ASD had complex comorbidities, including an anxiety disorder since early childhood, severe obsessive compulsive disorder (OCD), a personality disorder, a psychotic disorder and Tourette’s Syndrome. She had been sectioned for several years and lived on a closed in-patient ward. Her suffering consisted of continuous intrusive thoughts and compulsions. She described her life as a succession of misery, ignorance, doubt and struggle. She often felt lonely and sad. The RTE noted that it had been more difficult to treat her due to her ASD and psychotic vulnerabilities.

***Case 6*** *(2020-113, female, 50s, ID):* ***Suffering arising from a combination of somatic and psychiatric conditions***

A woman in her 50s, who had mild ID, suffered from spina bifida with total paralysis of her lower body. She also had osteoarthritis, spondylosis, kyphosis, chronic sacral pressure ulcers, renal dysfunction, tinnitus, tension headache, muscle atrophy and pain at her elbows, upper arms and shoulders. She was wheelchair-bound and lived in a care facility. In her youth, the patient had been affectively and emotionally neglected by her foster family and she had often felt unwanted, unsafe and vulnerable. In her adult life she had to move to a different care facility 15 times; the frequent changes of support workers and caregivers had demanded a lot from her adaptability. She had always felt rejected, no longer had faith in humanity, felt over-stimulated, and experienced her total dependence as an unbearable burden. She increasingly suffered from the physical effects of her infirmities, especially the pain in her arms and shoulders. She had no contacts or day-to-day activities that were meaningful to her. The patient didn't want to live like this anymore. Her physician thought that her disturbed neuro-cognitive and socio-emotional development made it too difficult for her to adapt and accept her limitations, and that the combination of somatic and psychological suffering was unbearable for her.
