# Supplementary material for: Euthanasia and physician-assisted suicide in people with intellectual disabilities and/or autism spectrum disorders: investigation of 39 Dutch case reports (2012–2021)
Source: BJPsych Open. 2023 May 23;9(3):e87. doi: 10.1192/bjo.2023.69 (PMC10228250; doi:10.1192/bjo.2023.69)
Supplement: Supplementary file 1 [file S2056472423000698sup001.zip › bjpsychopen-22-0506-20230420074417/graphic/EAS-ID-ASD_Table 4.docx]

**Table 4: Factors associated with suffering (N = 39)**

| **Factor** | **Number of cases** | **% of cases** |
| --- | --- | --- |
| Social isolation / loneliness | 30 | 77% |
| Physical symptoms *including:* | 27 | 69% |
| *Pain* | *19* | *49%* |
| *Tiredness* | *13* | *33%* |
| *Sleeping problems* | *9* | *23%* |
| Dependence | 24 | 62% |
| Lack of resilience or coping *(with the world, life and/or illness)* | 22 | 56% |
| Poor quality of life | 19 | 49% |
| Loss of hope | 18 | 46% |
| Lack of flexibility *including:* | 17 | 44% |
| *Difficulty adapting to change* | *12* | *31%* |
| *Rigid thinking / fixation* | *10* | *26%* |
| Oversensitive to stimuli | 10 | 26% |
| Loss of control | 6 | 15% |
| Negative self-image | 6 | 15% |
