# Supplementary material for: Euthanasia and physician-assisted suicide in people with intellectual disabilities and/or autism spectrum disorders: investigation of 39 Dutch case reports (2012–2021)
Source: BJPsych Open. 2023 May 23;9(3):e87. doi: 10.1192/bjo.2023.69 (PMC10228250; doi:10.1192/bjo.2023.69)
Supplement: Supplementary file 1 [file S2056472423000698sup001.zip › bjpsychopen-22-0506-20230420074417/suppl_data/EAS-ID-ASD_Supplement 1.docx]

**Supplementary File 1**

**Due care criteria for euthanasia and physician-assisted suicide in the Netherlands**

In 2002 The Netherlands passed a law, the *Termination of Life on Request and Assisted Suicide Act*, creating an exception to the Criminal Code. Under the Code ending another person's life or assisting a suicide was, and remains today, a criminal offence. The 2001 Act created an exception by providing that the Code would not apply where a physician terminated the life, or assisted the suicide, of a patient and where certain 'due care' criteria had been observed.

Under the Act, the physician must:

1. be satisfied that the patient’s request is voluntary and well-considered.
2. be satisfied that the patient’s suffering is unbearable, with no prospect of improvement;
3. have informed the patient about his situation and prognosis;
4. have come to the conclusion, together with the patient, that there is no reasonable alternative in the patient’s situation;
5. have consulted at least one other, independent physician, who must see the patient and give a written opinion on whether the due care criteria set out in (a) to (d) have been fulfilled;
6. have exercised due medical care and attention in terminating the patient’s life or assisting in his suicide.

*https://english.euthanasiecommissie.nl/due-care-criteria*
