# Supplementary material for: Euthanasia and physician-assisted suicide in people with intellectual disabilities and/or autism spectrum disorders: investigation of 39 Dutch case reports (2012–2021)
Source: BJPsych Open. 2023 May 23;9(3):e87. doi: 10.1192/bjo.2023.69 (PMC10228250; doi:10.1192/bjo.2023.69)
Supplement: Supplementary file 1 [file S2056472423000698sup001.zip › bjpsychopen-22-0506-20230420074417/suppl_data/EAS-ID-ASD_Supplement 2.docx]

**Supplementary file 2**

**Euthanasia & Assisted Suicide (EAS) notifications and published case reports**<https://www.euthanasiecommissie.nl/>

| **Year** | **Total number of EAS notifications** | **Total number of published case reports** | Number of published case reports  **Patient had ID** | Number of published case reports:  **Patient had ASD** | Number of published case reports:  **Patient had ID and ASD** |
| --- | --- | --- | --- | --- | --- |
| 2012 | 4,811 | 63 | 0 | 0 | 0 |
| 2013 | 4,829 | 107 | 2 | 1 | 0 |
| 2014 | 5,306 | 93 | 1 | 1 | 0 |
| 2015 | 5,516 | 82 | 1 | 0 | 0 |
| 2016 | 6,091 | 72 | 2 | 0 | 1 |
| 2017 | 6,585 | 97 | 0 | 2 | 0 |
| 2018 | 6,126 | 105 | 3 | 3 | 1 |
| 2019 | 6,361 | 102 | 2 | 4 | 0 |
| 2020 | 6,705 | 76 | 4 | 8 | 2 |
| 2021 | 7,666 | 130 | 0 | 1 | 0 |
| **TOTAL** | **59,996** | **927** | **15** | **20** | **4** |
