# Supplementary material for: Euthanasia and physician-assisted suicide in people with intellectual disabilities and/or autism spectrum disorders: investigation of 39 Dutch case reports (2012–2021)
Source: BJPsych Open. 2023 May 23;9(3):e87. doi: 10.1192/bjo.2023.69 (PMC10228250; doi:10.1192/bjo.2023.69)
Supplement: Supplementary file 1 [file S2056472423000698sup001.zip › bjpsychopen-22-0506-20230420074417/bjpsychopen-22-0506-manifest.html]

Manifest file for export bjpsychopen-22-0506-20230420074417


|  |  |
| --- | --- |
| File name: bjpsychopen-22-0506-20230420074417 | |
| Export Date: 20-Apr-2023 | |
| Output Format: XML (ScholarOne DTD) | |
| bjpsychopen-22-0506-20230420074417/doc/BJPsychOpen-22-0506.R3 clean.docx | Version 1.0 |
| bjpsychopen-22-0506-20230420074417/graphic/EAS-ID-ASD\_Table 1.docx | Version 1.0 |
| bjpsychopen-22-0506-20230420074417/graphic/EAS-ID-ASD\_Table 2.docx | Version 1.0 |
| bjpsychopen-22-0506-20230420074417/graphic/EAS-ID-ASD\_Table 3.docx | Version 1.0 |
| bjpsychopen-22-0506-20230420074417/graphic/EAS-ID-ASD\_Table 4.docx | Version 1.0 |
| bjpsychopen-22-0506-20230420074417/suppl\_data/EAS-ID-ASD\_Supplement 1.docx | Version 1.0 |
| bjpsychopen-22-0506-20230420074417/suppl\_data/EAS-ID-ASD\_Supplement 2.docx | Version 1.0 |
| bjpsychopen-22-0506-20230420074417/suppl\_data/UREC Letter for ITW - amended.pdf | Version 1.0 |
| bjpsychopen-22-0506-20230420074417/bjpsychopen-22-0506-metadata.xml |  |
| bjpsychopen-22-0506-20230420074417/s1.dtd |  |
| manifest.html | This document |
