# Supplementary material for: Euthanasia and physician-assisted suicide in people with intellectual disabilities and/or autism spectrum disorders: investigation of 39 Dutch case reports (2012–2021)
Source: BJPsych Open. 2023 May 23;9(3):e87. doi: 10.1192/bjo.2023.69 (PMC10228250; doi:10.1192/bjo.2023.69)
Supplement: Supplementary file 1 [file UREC Letter for ITW - amended.pdf › bjpsychopen-22-0506-20230420074417/suppl_data/UREC Letter for ITW - amended.pdf]

Kingston University London

T +44 (0)
D +44 (0)
E
www.kingston.ac.uk

Research and Impact
Tower Block
Penrhyn Road
Kingston upon Thames
Surrey KT1 2EE

6th April 2023

Dear Professor Tuffrey-Wijne

As Chair of the University Research Ethics Committee, I am writing to confirm that I have reviewed the research undertaken and can confirm that no ethical approval from Kingston University is required for this documentary analysis. The data was already in the public domain. No additional data was gathered and no data linkage has been undertaken.

I can confirm the background and methodology is as follows:

- Every year, the Dutch government publishes a number of anonymised case reports of people who have received Euthanasia or Assisted Suicide (EAS) by a physician. The reporting of all EAS cases is a legal requirement in the Netherlands. The case reports are scrutinised by a committee, who publishes a selection of anonymised case reports in an Annual Report, plus a wider selection online. This is freely available to the public, see here: https://english.euthanasiecommissie.nl/
- There are 927 such reports publicly available (in Dutch). As a native Dutch speaker, you were able to search this database for cases of people who had intellectual disabilities and/or autism. You found 39 such case reports. Here is an example of just one such case report https://www.euthanasiecommissie.nl/uitspraken/publicaties/oordelen/2021/2021-21-tm-2021-40/oordeel-2021-26
- You undertook a content analysis of the texts of all these 39 reports. The results are found in your paper submitted to BJPsych Open – I have reviewed the manuscript which is at the point of being accepted for publication.
- No additional data was collected, and the case reports were not combined with any other data sets. You have simply used and cited the data that were made available to the public (including researchers) by the Dutch government.

Therefore I can confirm, as Chair Research Ethics Committee that your study reported in the submission to BJPsych Open does not need research ethics approval.

With best wishes

Cilla

Professor Priscilla Harries, PhD, MSc, DipCOT, FHEA, FRCOT

Chair, University Research Ethics Committee

Kingston University London

Email: p.harries@kingston.ac.uk

Twitter: https://twitter.com/Cilla_Harries

Research gate: https://www.researchgate.net/profile/Priscilla_Harries

LinkedIn: https://uk.linkedin.com/in/professor-priscilla-harries-10429753
